# Watchman vs. Amulet for Left Atrial Appendage Closure: Current Evidence and Future Perspectives

**DOI:** 10.3390/jcm13164651

**Published:** 2024-08-08

**Authors:** Marco Frazzetto, Claudio Sanfilippo, Giuliano Costa, Claudia Contrafatto, Chiara Giacalone, Salvatore Scandura, Giuseppe Castania, Jessica De Santis, Maria Sanfilippo, Maria Elena Di Salvo, Corrado Tamburino, Marco Barbanti, Carmelo Grasso

**Affiliations:** 1 Division of Cardiology, A.O.U. Policlinico “G. Rodolico San Marco”, 95123 Catania, Italy; cla.sanfi24@gmail.com (C.S.); giulianocosta90@gmail.com (G.C.); claudiacontra@icloud.com (C.C.); chiaragiacalone96@libero.it (C.G.); salvatore.scandura@tin.it (S.S.); giuseppe.castania16@gmail.com (G.C.); jessicadesantis3@gmail.com (J.D.S.); sanfilippomariap@gmail.com (M.S.); mari.disalvo@gmail.com (M.E.D.S.); corradotamburino@centrocuore.it (C.T.); melfat75@gmail.com (C.G.); 2 Faculty of Medicine and Surgery, Università degli Studi di Enna “Kore”, 94100 Enna, Italy; mbarbanti83@gmail.com; 3 Division of Cardiology, Ospedale Umberto I, ASP 4 di Enna, 94100 Enna, Italy

**Keywords:** atrial fibrillation, left atrial appendage closure, Watchman, Amulet

## Abstract

Left atrial appendage closure (LAAC) is a crucial intervention for stroke prevention in patients with non-valvular atrial fibrillation who are unsuitable for long-term anticoagulation. Amulet and Watchman are the most implanted devices worldwide for performing LAAC, and the aim of this review is to provide a comprehensive comparison focusing on their efficacy, safety, and short- and long-term outcomes. The Watchman device, the first to gain FDA approval, has been extensively studied and demonstrates significant reductions in stroke and systemic embolism rates. The Amulet device, a newer alternative, promises enhanced design features for more efficient appendage sealing. Current data highlight that both devices offer similar efficacy and safety for LAAC. While the two devices differ in terms of intraprocedural complication rates, they offer similar short- to long-term outcomes in terms of peri-device leaks, device-related thrombosis, and mortality. Both devices are indicated for patients who are unable to tolerate OAC, given their similar risk and safety profiles. Newer clinical studies are directed at establishing the efficacy of both devices as the primary method for stroke prevention in AF as an alternative to OAC.

## 1. Introduction

Atrial fibrillation (AF) is the most common cardiac arrhythmia, associated with a five-fold increase in risk of cardioembolic stroke [1]. Large-scale studies have estimated an overall prevalence of 0.9% in the US, increasing to 3–5% in people older than 65 years, and to 10% or higher in people over 80 years of age [2]. Additionally, AF imposes an important cost burden on healthcare systems due to its morbidity, mortality, and therapeutic interventions. A large UK-based survey revealed that the direct cost of managing AF increased from 0.6–1.2% of the total National Health Service budget in 1995 to 0.9–2.4% by 2000 [3].

Many patients with AF develop bleeding complications due to long-term treatment with oral anticoagulation (OAC), which often requires its discontinuation. Percutaneous left atrial appendage closure (LAAC) is a valid therapeutic option in patients with high bleeding risk or contraindication to OAC to prevent cardioembolic events.

The aim of LAAC is to exclude from circulation the left atrial appendage (LAA), which is the main source of cardiac thrombi in patients with non-valvular atrial fibrillation [4]. For many years, the class of indication to perform LAAC has been IIB in the European and American guidelines [5,6], which is probably because some authors consider that pivotal studies on LAAC did not meet non-inferiority properly. Recently, the new American guidelines on AF in consideration of the amount of positive data on LAAC decided to promote the procedure to class IIA in patients with contraindication to anticoagulation; for patients at high bleeding risk, the indication remains in class IIB [7].

Initially, the Watchman 2.5 (Boston Scientifics) and Amplatzer Cardiac Plug (Abbott Vascular) devices were the first-generation devices most used worldwide for percutaneous LAAC. Recently, their iterations Watchman FLX and Amulet were approved by the FDA in 2020 and 2021, respectively. More recently, the latest iteration Watchman FLX PRO has been released.

In the last decade, randomized controlled trials and multicenter registries confirmed the safety and effectiveness of both devices (Table 1) [8]. The aim of this review is to analyze the similarities and differences between Watchman and Amulet by summarizing the available evidence regarding the procedural and long-term outcomes of LAAC using these two devices.

## 2. Device Specification

**Figure 1:**
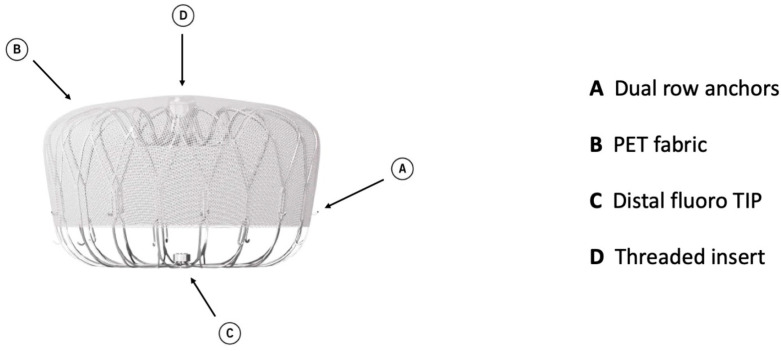
Watchman FLX device construction.

**Figure 2:**
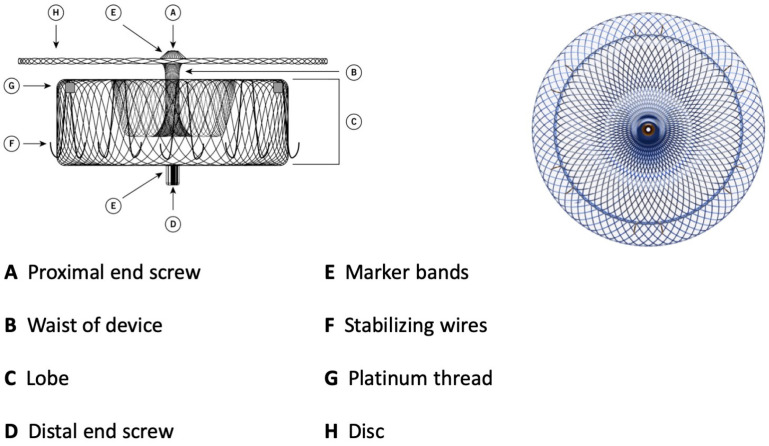
Amulet device construction.

Watchman 2.5 had a self-expanding nitinol structure with a porous covering on the proximal face, fixation barbs to minimize embolization, and a permeable polyester fabric cover. The device was available in five sizes: from 21 mm to 33 mm. The Watchman FLX device is the iteration of Watchman 2.5 and brings a range of improvements. It is available in five sizes (20, 24, 27, 31, and 35 mm), which allows the treatment of a broader range of LAA ostia, covering diameters from 14 mm to 31.5 mm. The device comprises a self-expanding nitinol frame with 18 peripheral J-shaped fixation anchors (two rows of nine hooks) and a polyester fabric covering the atrial-facing surface (Figure 1). The atraumatic closed distal end was designed to reduce the risk of LAA perforation, provide control and stability of the deployment, and allow full and partial recapture and redeployment. The 18- rather than 10-strut design permits a better conformation and fixation to the LAA ostium, reducing peri-device leaks (PDLs). The recessed threaded insert in the center of the proximal face and the PET fabric extended more distally to decrease the amount of exposed metal volume to potentially improve endothelization and reduce device-related thrombus (DRT). Recently, a novel iteration has been released, the WATCHMAN FLX Pro. This device incorporates three novel and unique features: new HEMOCOAT Technology to improve the healing process, radiopaque markers that provide more precise placement, and a new 40 mm size to cover up to 36 mm ostia diameter. The device is preloaded in a 12 Fr delivery catheter. The three iterations of the access system, currently available, are Watchman TrueSeal^TM^, Watchman FXD Curve^TM^, and Watchman TrueSteer^TM^. TrueSeal^TM^ is a 14 Fr catheter available in single, double, and anterior curve configurations. FXD Curve^TM^ Access Sheath is a 15 Fr catheter available in single- and double-curve shapes; the double curve optimizes the secondary curve height, and with the enhanced torque transmission, this allows a wider range of anterior anatomies to be reached compared to the double- and anterior curve shapes of previous sheaths. Watchman TrueSteer^TM^ is a bi-directional steering catheter which provides a broad range of motion, allowing access to the greatest range of anatomies (Table 2). Amplatzer Amulet (Abbott Vascular) is a self-expanding device made of nitinol with a distal lobe and a proximal disc, connected by an articulating waist. Polyester patches are sewn into both the lobe and disc to facilitate occlusion (Figure 2).

The lobe has stabilizing wires to improve device placement and retention. The device has threaded screw attachments at each end for connection to the delivery and loading cables. It has radiopaque markers at each end and at the stabilizing wires that permit visibility during fluoroscopy. As for the Watchman device, it can be easily recaptured and repositioned. The Amulet is available in six different sizes, covering LAA ostial diameters between 11 and 31 mm and depths ≥ 12 mm. The TorqVue™ 45–45° sheath is the default sheath for both ACP and Amulet devices. The new Amplatzer Steerable Delivery Sheath (ASDS) is designed to improve the ease of use in both simple and challenging left atrial appendage anatomies, it has a flexible design capable of bidirectional movement from 0 to 120° to ensure coaxial alignment with the ostium (Table 2). The procedural steps for both devices are essentially the same as well as the learning curve of the operators that correlates with outcomes [9,10]. A crucial difference lies in the deployment and release of two devices depending on the two different sealing mechanisms. The Watchman, a single-occlusive mechanism, is released at the ostium, while the Amulet, a dual-occlusive mechanism, is released at the landing zone (1 cm deeper than ostium) considering the presence of the disc. The Watchman and Amulet device release criteria are summarized in Table 3.

## 3. Periprocedural Outcomes

The safety and effectiveness of the Watchman device were originally assessed in two randomized controlled trials. The landmark PROTECT AF trial (WATCHMAN Left Atrial Appendage System for Embolic Protection in Patients with Atrial Fibrillation) showed an implant success rate of 90.9% and device-related or procedural stroke of 1.1%. The incidence of intraprocedural pericardial effusion and major bleeding was 4.8% and 0.6%, respectively. The device embolization (DE) rate was 0.4% [11].

The subsequent PREVAIL trial (Prospective Randomized Evaluation of the Watchman LAA Closure Device In Patients With Atrial Fibrillation vs. Long-Term Warfarin Therapy) reported remarkable improvement in implant success rate to 95.1% with the rate of procedural and device-related stroke decreasing from 1.1% to 0.4%. Pericardial effusion requiring surgical repair decreased to 0.4% in such a trial. No difference was observed in terms of DE across the studies [12].

PRAGUE 17 is a multicenter, prospective, open-label, randomized, non-inferiority trial that investigated the effectiveness of LAAC with different devices compared to direct oral anticoagulants (DOACs), mainly apixaban. The device implanted was Amulet, Watchman, or Watchman FLX in 61.3%, 35.9%, and 2.8% of cases, respectively. The rate of periprocedural complications, including pericardial infusion, DE, vascular complications, and device- or procedure-related death, was 2.1%. In 96.8% of the patients, the device was successfully implanted [13].

The PINNACLE FLX trial, a prospective, single-arm, multicenter study, evaluated the outcome of the new-generation device, Watchman FLX, in 400 patients with high thromboembolic risk not eligible for OAC. The implant success rate was 98.8%, and the primary safety endpoint, a composite of death, ischemic stroke, systemic embolism (SE), and device- or procedure-related events requiring open cardiac surgery or major endovascular intervention, occurred in 0.5% of patients at 7 days. The safety results compare favorably with the those of the previous generation device used in CAP, PREVAIL, and CAP2 studies [14].

Different subsequent studies have confirmed the safety and efficacy of the Watchman FLX device in a real-world setting [15,16].

In a single center registry, Saad et al. compared Watchman 2.5/Watchman FLX vs. Amulet and reported that the Amulet group had more periprocedural complications (2.7% vs. 10.6%) and more major bleeding complications (0% vs. 5.3%) [17].

The SWISS-APERO trial demonstrated a higher rate of pericardial effusion (17.1% vs. 6.4%) and bleeding (25.2% vs. 13.6%), mostly consisting in pericardial effusion, with Amulet compared with Watchman 2.5/FLX in patients undergoing LAAC. The implant success rate (94.6% vs. 97.3%) was similar in both groups [18].

**Figure 3:**
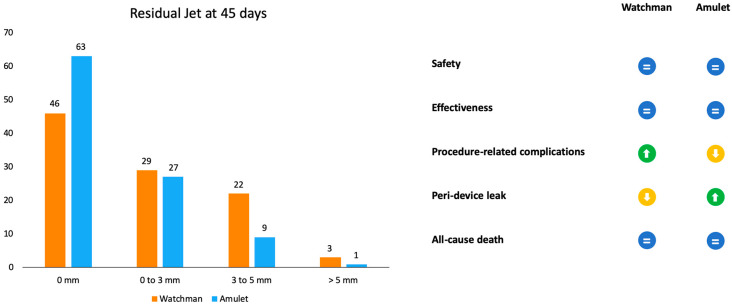
Representation of outcomes in residual jet at 45 days and advantages or disadvantages of the two devices according to the Amulet IDE trial.

Amulet IDE was the first randomized trial to have compared Amulet and Watchman devices (Figure 3). Patients were randomly assigned to receive one of the two devices (934 with Amulet vs. 944 with Watchman 2.5/Watchman FLX). The primary safety endpoint was a composite of procedure-related complications, all-cause death, and major bleeding. The procedural success rate was similar between the Amulet, 97.2%, and Watchman, 95.3%, devices. Procedure-related complications were higher for the Amulet occlude (4.5%) in comparison to the Watchman device (2.5%), mainly driven by pericardial effusion and DE [19].

A recent meta-analysis evaluated the efficacy and safety of the new-generation Watchman FLX device compared to the Watchman 2.5 device. It demonstrated that the Watchman FLX device was associated with greater procedural success and a greater reduction in periprocedural complications including mortality, major bleeding, DE, and pericardial effusion [20].

To note, most LAAC procedures worldwide are performed under TEE guidance. Recently, a minimalistic approach with pre-procedural CCTA and Intracardiac echocardiography (ICE) guidance without sedation/intubation has been taking place. Such an approach has shown promising results in terms of patient safety and LAAC efficacy with both devices in experienced centers [21,22,23,24].

The current data present in the literature highlight that the Amulet device is associated with more periprocedural complications, mainly pericardial effusion, compared to the Watchman device. These differences may be attributable to the early implantation experience with the Watchman device, the introduction of the Amulet device later at the end of 2016, and the cup/disc shape of the Amulet compared with Watchman “free disc” design, which could increase the incidence of pericardial effusion events during and after implantation.

## 4. DRT and PDLs

**Figure 4:**
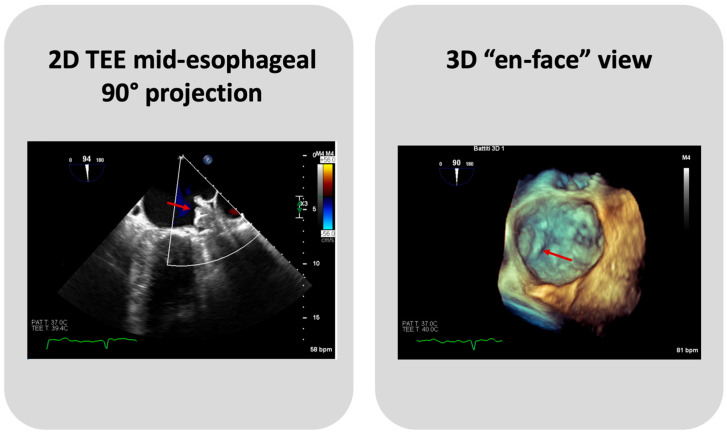
A case of DRT on Watchman FLX device. The arrows indicate the thrombus on device.

TEE or Cardiac CT assessment at 45 days are essential to evaluate PDLs, DE, and DRT (Figure 4). Until recently, data on the clinical significance and the optimal management of PDLs after LAAC have been limited, and an arbitrary cut-off of a 3 or 5 mm leak diameter was utilized, for commercially available devices, to define clinically significant leaks. Nevertheless, recent data show that even small PDLs have a negative impact on outcomes, and hence, techniques to mitigate and manage PDLs also need to be further investigated [25]. The association between DRT and thromboembolic events, as plausible as it may seem, has been inconsistently demonstrated in the literature [13]. In PROTECT AF and PREVAIL trials, DRT was associated with a significant increase in ischemic stroke or SE (HR: 3.9; 95% CI: 2.3–6.8; *p* < 0.001) [26]. In several DRT registries, a similar association between DRT and 2-to-4-fold increases in ischemic events have been documented [27,28]. However, in the EWOLUTION Registry, no significant difference was found in the annual rates of stroke or transient ischemic attack or SE between patients with or without DRT (1.7% vs. 2.2% per year, respectively; *p* = 0.80) [29], nor did the Amulet IDE study demonstrate statistically significant associations between DRT and ischemic stroke or SE (3.1% vs. 2.6% with vs. without DRT) [30]. Of note, such data should be cautiously interpreted, as the absence of DRT at the time of ischemic events does not preclude its presence prior to the event. The PROTECT AF trial found that the rates of PDLs > 3 mm and DRT were 13.3% and 3.4%, respectively, after 45 days from LAAC with the Watchman 2.5 device [11]. The subsequent PREVAIL trial showed that at 45-day, 6-month, and 12-month follow-ups after successful implantation, 92.2%, 98.3%, and 99.3% of patients, respectively, were able to discontinue warfarin, meaning that none of these patients had an incomplete closure of the LAA, a PDL > 5 mm, or a definite visible large thrombus on the device. Additionally, DE was infrequent, occurring only in two patients [12]. The results from the EWOLUTION registry, a multicenter, prospective, nonrandomized cohort study including over 1000 patients treated with the Watchman device, provided 1-year follow-up data revealing that DRT was detected by TEE in 3.7% of patients [29]. A reduction in DRT and PDLs was observed in the PINNACLE FLX trial. The incidence of DRT was 1.75% at a 12-month follow-up, whilst the rate of PDLs > 5 mm and DE was 0% at both 45-day and 1-year follow-ups. This important finding may be due to the operation design changes in the Watchman FLX device, like the decreased height, the presence of two rows of anchors, and the ability to fully recapture and reposition the device, allowing for optimal deployment. Of note, the rate of PDLs less than 5 mm at 45-day and 1-year follow-ups was 17.2% and 10.5%, respectively [14]. A multicenter prospective global Amulet observational study showed that following LAAC with the AMPLATZER Amulet device, DRT was infrequently observed with a rate of 1.7% patients/year. Eighty-two percent of these patients had a suboptimal implantation, meaning that the left upper pulmonary vein ridge was not covered by the Amulet disc. DRT presence increases the risk of clinically relevant events as compared with patients with absence of DRT, despite the majority of DRT patients not reporting a thromboembolic events [31]. The ORIGINAL Registry (saxOnian RegIstry analyzinG and followINg left atrial Appendage cLosures), a multicenter prospective clinical registry including nine hospitals in the Federal State of Saxony in Germany evaluated the efficacy and safety of LAAC with the AMPLATZER Amulet and Watchman 2.5. The TEE outcome data revealed DRT after 6 weeks in 4.6% of patients of the Watchman group and no thrombus in the Amulet group. Regarding PDLs, TEE showed a residual PDL ≥ 5 mm in 1.4% of patients in the Watchman group, and no PDLs in the Amulet group. However, these differences are numerically but not statistically significant [32]. In the Amulet-IDE trial, at 18 months, the DRT rate was similar between groups (3.3% with Amulet vs. 4.5% with Watchman 2.5/FLX). The rate of 0–3 mm PDLs at a 45-day follow-up was similar between Amulet and Watchman 2.5/FLX (27% and 29%, respectively). Patients that received the Watchman device showed a higher rate of 3–5 mm PDLs than the Amulet group (22% vs. 9%). Concerning PDLs > 5 mm, Watchman 2.5/FLX showed a rate of 3% vs. 1% of Amulet. The lower rates of leaks could be explained by the double-sealing mechanism of the Amulet device [19]. A subanalysis of the Amulet-IDE trial revealed that PDLs > 3 mm were associated with a higher, but not statistically significant, risk for ischemic stroke or SE at 18 months [33]. Dukkipati et al., using combined data from the PROTECT-AF, PREVAIL, and CAP2 (Continued Access to PREVAIL) trials, assessed patients with successful device implantation for PDLs by means of TEEs performed at 45 days and 1 year, analyzing five-year outcomes as a function of the absence or presence of PDLs ≤ 5 mm. Compared to the 45-day assessment, the 1-year TEE revealed that the rate of PDLs ≤ 5 mm reduced from 38.3% to 27.7%. The presence of PDLs ≤ 5 mm at 1 year, but not at 45 days, was associated with an increased 5-year risk of ischemic stroke or SE (adjusted HR: 1.94; 95% CI: 1.15–3.29; *p* = 0.014), driven by an increased risk of non-disabling stroke, whilst the disabling or fatal stroke rates were similar [34]. A study by Saw et al. analyzed patients who underwent LAAC with the Watchman or Amulet device and had CCTA and TEE post-LAAC. CCTA was performed at a mean of 105.2 days, and TEE at a mean of 124.9 days post-LAAC. LAA patency was observed in 52/100 (52%), with 45 (86.5%) via PDLs and 7 (13.5%) through fabric leaks, whilst PDLs were only observed in 35/102 (34.3%) via TEE. Only one case of DRT was detected, which was observed via both TEE and CCTA [35]. The SWISS-APERO (Comparison of Amulet vs. Watchman/FLX Device in Patients Undergoing Left Atrial Appendage Closure) randomized controlled trial compared data about patients who underwent LAAC with Amulet and Watchman/Watchman FLX. At 45 days, the PDL rate via TEE was higher with Watchman/Watchman FLX than with Amulet (27.5% vs. 13.7%, *p* = 0.020), but none was <5 mm. DRT was detected in 0.9% of patients with Amulet and 3.0% of patients with Watchman/Watchman FLX via CCTA, and in 2.1% and 5.5% of patients via TEE, respectively [18]. A pre-clinical study compared the thrombogenicity and endothelial coverage after LAAC between the conventional uncoated WATCHMAN FLX and the more recent WATCHMAN FLX PRO, using experimental in vitro and animal models. The results from this study showed a significantly lower percentage of DRT at 45 days in canines implanted with FLX-PRO than those implanted with FLX (0% vs. 50%; *p* < 0.05) [36]. These results highlighted the reduced thrombogenicity, lower inflammation, and albumin-mediated reduction in platelet binding associated with the fluoropolymer coatings of the novel Watchman iteration.

## 5. Long-Term Outcomes

The evaluation of long-term outcomes after LAAC is focused on all-cause mortality, cardiovascular death, ischemic and hemorrhagic stroke, major and minor bleeding, and nonprocedural major bleeding, and all factors are crucial to define procedural safety and effectiveness over time compared to anticoagulant therapy.

Patients enrolled in the PROTECT AF and PREVAIL trials were included in a patient-level meta-analysis which showed, at a 5-year follow-up, similar rates for the primary composite endpoint of stroke, SE, or cardiovascular/unexplained death in Watchman and warfarin groups (hazard ratio [HR]: 0.82; 95% CI: 0.58 to 1.17; *p* = 0.27). The ischemic stroke/SE rate was numerically higher with LAAC, not reaching statistical significance (HR: 1.71; *p* = 0.080). However, LAAC induced a reduction in hemorrhagic stroke (HR: 0.20; 95% CI: 0.07–0.56; *p* = 0.0022), disabling/fatal stroke (HR: 0.45; 95% CI: 0.21–0.94; *p* = 0.034), and all-cause/cardiovascular mortality [37].

The findings from the PRAGUE-17 trial, which compared LAAC with DOACs in high-risk patients, demonstrated that LAAC was non-inferior to DOACs in preventing major AF-related cardiovascular, neurological, and bleeding events. No difference appeared between groups for the primary composite endpoint of cardioembolic events, cardiovascular death, clinically relevant bleeding, or procedure-/device-related complications (sHR: 0.84; 95% confidence interval [CI]: 0.53 to 1.31; *p* for non-inferiority = 0.44) at a 1-year follow-up [13]. After a 3.5-year median follow-up, LAAC was confirmed to be non-inferior to DOACs (*p* for non-inferiority = 0.006) for the primary composite endpoint of cardioembolic events, cardiovascular death, clinically relevant bleeding, or procedure-/device-related complications. Additionally, the LAAC strategy significantly reduced the nonprocedural clinically relevant bleeding and the two arms did not differ regarding each remaining component of the composite endpoint [38].

In a meta-analysis comprising the three randomized controlled trials PREVAIL, PROTECT AF, and PRAGUE 17, positive outcomes were observed in terms of long-term results for LAAC. The incidence of all strokes or SE did not significantly differ between LAAC and OAC groups (RR: 0.98; 95% CI: 0.65 to 1.48; *p* = 0.92). Although it seemed to be a slightly higher rate of ischemic stroke/SE with LAAC, this difference was not statistically significant. Conversely, a significantly elevated risk of hemorrhagic strokes (RR: 0.22; 95% CI: 0.08 to 0.58; *p* = 0.002), cardiovascular death (RR: 0.65; 95% CI: 0.44 to 0.95; *p* = 0.03), and all-cause death (RR: 0.78; 95% CI: 0.62 to 0.99; *p* = 0.04) was noted in the OAC group. Concerning major bleeding, no significant intergroup difference was observed; instead, a reduction in non-procedure-related major bleeding rate was evident in the LAAC group (RR: 0.53; 95% CI: 0.38 to 0.74; *p* = 0.0002). The benefit for the mortality rate appears to be driven by a 78% reduction in hemorrhagic stroke and 47% decrease in non-procedure-related bleeding [39].

The one-year outcomes from the SWISS-APERO trial, an open-label, multicenter, randomized clinical study comparing the Amulet device to the Watchman 2.5/FLX, revealed that the devices demonstrated similar results regarding the composite of cardiovascular death/ischemic stroke/SE (9.5% Amulet vs. 10.2% Watchman 2.5/FLX) and the composite of ischemic stroke/SE (2.7% Amulet vs. 3.8% Watchman 2.5/FLX). Additionally, the rates of bleeding events were similar (40.8% Amulet vs. 31.4% Watchman 2.5/FLX; HR: 1.46 [95% CI: 0.93–2.28]; *p* = 0.098) [40].

The Amulet-IDE trial showed the non-inferiority of the Amulet compared to Watchman device in terms of the primary safety endpoint at 12 months, reporting similar major bleeding (10.6% Amulet vs. 10.0% Watchman) and all-cause death rates between groups. Also, primary effective endpoints were similar, with comparable ischemic stroke (2.5% Amulet vs. 2.7% Watchman) and SE rates (0.3% Amulet vs. 0.2% Watchman at an 18-month follow-up [19].

The WATCHMAN 2.5 and ACP/Amulet were compared using data from the prospective, multicenter LAARGE (Left-Atrium-Appendage Occluder Register—GErmany) registry.

Both devices showed comparable results at one year for the composite endpoint of death, stroke, or SE (12.0% and 12.9%, respectively) [41].

A study-level metanalysis by Bing et al. revealed similar long-term outcomes of stroke (OR: 1.24, 95% [CI]: 0.92–1.67, *p* = 0.17, I2 = 0) and SE (OR: 1.10, 95% CI: 0.51–2.35, *p* = 0.81) for the Watchman and ACP/Amulet devices. This metanalysis also found rates regarding all-cause death (OR: 0.97, 95% CI: 0.80–1.18, *p* = 0.77), cardiac death (OR: 0.99, 95% CI: 0.77–1.29, *p* = 0.96) and major bleeding (OR: 1.18, 95% CI: 0.98–1.43, *p* = 0.08), corroborating the similarity of such devices in terms of safety endpoints [42].

The PINNACLE FLX study disclosed the following results at the 12-month mark: the primary effectiveness endpoint, defined as closure of the LAA with a jet size < 5 mm on TEE, was attained in every case, demonstrating a 100% success rate. The incidence rates at 12 months were 6.6% for all-cause mortality, 2.6% for all strokes, and 7.9% for major bleeding [14].

A systematic review and meta-analysis conducted by Najim et al., aimed at comparing the safety and efficacy of the Watchman 2.5 to the newer Watchman-FLX device in clinical practice, revealed a statistically significant reduction in device embolism [OR 0.35, 95% CI 0.18–0.70, *p* = 0.02], major bleeding [0.57, 95% CI 0.51–0.64, *p* < 0.01; I2 = 0%], pericardial effusion [OR 0.33,95% CI 0.26–0.41, *p* < 0.01], mortality [OR 0.52 95% CI 0.51–0.54, *p* < 0.01], and an increase in procedural success [OR 7.49, 95% CI 1.98–28.26, *p* = 0.02] favoring the newest generation device. However, the relatively short follow-up period of three months represents a limitation of this study, as it could potentially obscure late complications [20].

## 6. Anti-Thrombotic Therapy

An unresolved issue in the LAAC field is which antithrombotic therapy following the procedure could be optimal to balance the bleeding and thromboembolic risk.

The FDA approval of the Watchman 2.5 device in 2015, based on the PROTECT AF and PREVAIL trial, incorporated the use of a standardized protocol to adopt after LAAC procedure [11,12]. It included antithrombotic therapy, imaging, and follow-up. Patients took Warfarin and aspirin (81–325 mg) for 45 days after LAAC, and after this period, they underwent TEE to assess the absence of PDLs < 5 mm or DRT, essential to decide whether to discontinue warfarin and continue with a dual antiplatelet therapy with Clopidogrel 75 mg daily and aspirin daily until 6 months, then aspirin lifelong. If a 45-day follow-up showed PDLs or DRT, patients continued on warfarin and aspirin.

However, warfarin is vulnerable to drugs and food and requires frequent monitoring for an international normalized ratio that contributes to low compliance for patients. An alternative to warfarin is DOACs, which are the first-choice treatment in AF patients without contraindications.

The following studies analyzed alternative postoperative regimens, showing that DAPT alone may be sufficient to prevent device-related thrombus and stroke.

The EWOLUTION registry, which evaluates real-life clinical outcomes, analyzed patients who underwent LAAC with the Watchman 2.5 device and were treated with different post-procedure regimens. Upon discharge, 16% of patients were prescribed Vitamin K Antagonists (VKA), 11% DOACs, 60% Dual Antiplatelet Therapy (DAPT), and 7% Single Antiplatelet Therapy (SAPT), and 6% received no anticoagulation. Data from the initial three months of follow-up revealed no statistically significant correlation between DRT, stroke, and bleeding rates and the post-procedure regimen administered [43]. At 1 year, 84% of active patients were on SAPT or no anticoagulation [44]. The results at the 2-year mark showed DRT in 4.1% of patients, yet no statistical relationship was established between DRT and the type of anticoagulation used, and no significant correlation was observed between stroke, transient ischemic attack, SE rates, and the type of medication administered [34]. Furthermore, this registry illustrated a nearly halved major bleeding rate compared to the anticipated rate with VKA. A specific emphasis on antithrombotic medication for higher-risk cohorts with prior ischemic and hemorrhagic stroke, as well as major bleeding, was undertaken in the EWOLUTION registry. Patients with a history of hemorrhagic stroke or major bleeding were discharged with lower rates of OAC compared to the overall population, as SAPT/no therapy or DAPT was preferred for these patients. Despite the decreased use of OAC, patients with prior hemorrhagic stroke exhibited a significant reduction in the risk rate of ischemic stroke/TIA/SE, along with a 67% decrease in major bleeding events [45].

These findings suggest that post-LAAC therapy without warfarin, opting instead for direct DAPT or DOAC at discharge, demonstrated non-inferiority to warfarin concerning thromboembolic outcomes.

Data from the NCDR LAAO registry about 31,994 patients treated with Watchman devices and divided into subgroups discharged with different antithrombotic therapies revealed statistically different rates of any adverse events within the 45 days after the procedure. These rates were highest among patients treated with warfarin and aspirin (5.7%), followed by DAPT (5.6%), DOAC and aspirin (5.3%), warfarin (4.0%), and DOAC (3.8%) (*p*-value < 0.0001). Similar differences were reported for any major adverse events rates with decrescent rates reported in the same order. Although DAPT was associated with a significantly higher unadjusted rate of device-related thrombus compared to other treatment groups, no significant differences in rates of readmission, any stroke or TIA, and PDLs > 5 mm were found [46].

Similar results were found in a propensity-matched analysis including all patients who underwent LAAC with the Watchman device from the randomized PROTECT-AF, PREVAIL, CAP, CAP2, and ASAP trials and the EWOLUTION registry, which found no significant difference in the safety and efficacy endpoint between the OAC and antiplatelet therapy APT group, although DRT was significantly more frequent in the APT group (3.1%) than in the OAC (1.4%) group [47].

Unlike Watchman 2.5, the Amulet device did not require an OAC regimen after the procedure, as stated in the AMULET IDE trial. In the Amulet group, participants were discharged on either dual antiplatelet therapy or aspirin plus OAC according to investigator discretion. In the Watchman group, participants were discharged on aspirin and warfarin. After 45 days, both groups were instructed to begin the same medication regimen (DAPT until 6 months followed by aspirin monotherapy indefinitely). Overall, 75.7% of patients with the Amulet occluder were discharged on aspirin and clopidogrel, while 82% were discharged on warfarin plus aspirin [19]. At a 3-year follow-up, a significantly higher percentage of patients were free of oral anticoagulation usage with Amulet (96.2%) vs. Watchman (92.5%) (*p* < 0.01). The clinical outcomes were comparable for the composite of ischemic stroke or SE (5.0% vs. 4.6%; *p* = 0.69) and in terms of major bleeding (16.1% vs. 14.7%; *p* = 0.46).

A single-center, prospective, non-randomized study found that LAAC with the ACP or Amulet was safely performed with ASA monotherapy postoperative, showing a 61% stroke risk reduction compared to the predicted rate based on the CHA2DS2-VASc score. Moreover, the annual risk of major bleeding was reduced by 57% [48].

In a recent study by Kramer et al., 431 high-bleeding risk patients who underwent LAAC with the Amplatzer Cardiac Plug (ACP) or Amulet, were discharged on SAPT with either acetylsalicylic acid (72.9%) or clopidogrel (5.1%). Their DRT rate on an 8-week follow-up imaging was comparable to non-SAPT patients (1.5% SAPT vs. 2.7% non-SAPT). Additionally, ischemic stroke was comparable to non-SAPT patients, while the observed major bleeding rate was lower [49].

The next-generation Watchman FLX was approved by the FDA for DAPT immediate use post-implant, allowing one to choose between two different post-procedure therapeutic regimens. The first one is the traditional OAC with aspirin for the initial 45 days followed by 6 months of DAPT and then aspirin for a lifetime. The second option involves starting DAPT immediately post-implantation up to 6 months followed by aspirin lifelong. If the 45-day follow-up showed PDLs > 5 mm, patients continued OAC with aspirin until PDLs were <5 mm. A study analyzed data from the NCDR LAAO Registry, focusing on 49,968 patients undergoing Watchman FLX implantation between August 2020 and September 2021. The objective was to compare different post-procedure regimens at discharge (DAPT, DOAC/aspirin, or warfarin/aspirin). Data were analyzed using a propensity score matching analysis, which showed no statistically significant differences for 45-day composite endpoint rates (death, stroke, major bleeding, and SE) between groups (DAPT 3.44% vs. DOAC/aspirin 4.06%; *p* = 0.13 and DAPT 3.23% vs. warfarin/aspirin: 3.08%; *p* = 0.75). Furthermore, each component of the composite endpoint did not exhibit differences among the different groups. The rate of major bleeding was slightly higher in DOAC/aspirin compared to DAPT patients (DAPT 2.48% vs. DOAC/aspirin 3.25%, *p* = 0.004), thus reinforcing the notion that discharging the patient on DAPT instead of OAC may represent an advantage, especially in high-bleeding-risk patients [50].

In a study by Della Rocca et al., an alternative treatment regimen was investigated. The study analyzed data from a cohort of patients who underwent Watchman LAAC, dividing them into two groups: one group received standard antithrombotic therapy, comprising 45 days of full-dose DOAC combined with aspirin 81 mg, followed by a transition to aspirin and clopidogrel for 6 months, and then monotherapy with aspirin lifelong; the second group was treated with half-dose DOAC alongside aspirin 81 mg for 45 days, followed by lifelong half-dose DOAC monotherapy. The composite endpoint of DRT, thromboembolic events, and major bleeding was found to be lower in patients treated with half-dose DOAC compared to those on SAT (9.5% SAT vs. 1% half-dose DOAC; HR: 9.8; 95% CI: 2.3–40.7; *p* = 0.002). The DRT incidence was significantly lower with half-dose DOAC compared to SAT (3.4% SAT vs. 0.0% half-dose DOAC), as was the rate of major bleeding (3.9% vs. 0.5%; HR: 7.9; 95% CI: 1.1–49.9; *p* = 0.04) [51].

Currently, there are no clear guidelines about the best post-LAAC antithrombotic regimen, and the choice is often tailored to patient conditions, balancing thrombotic and hemorrhagic risk.

## 7. Ongoing Trials and Future Directions

While several clinical trials, including PROTECT AF and PREVAIL have provided pivotal evidence supporting the safety and efficacy of LAAC, the landscape of AF management continues to evolve rapidly.

Many randomized trials on percutaneous LAAC using Amulet or Watchman devices are ongoing (Table 4) [8].

Recognizing the importance of evidence-based medicine plays a crucial role in shaping clinical practice and refining treatment strategies [8].

Currently, LAAC is indicated as a second-line intervention for stroke prophylaxis in individuals unable to tolerate or unwilling to adhere to OAC therapy. CATALYST (NCT04226547) and CHAMPION-AF (NCT04394546) aim to evaluate LAAC utilizing Amulet and Watchman FLX, respectively, as a first-line therapy compared to DOAC in patients eligible for long-term DOAC therapy.

Other trials assess the role of LAAC in special populations. In particular, the OPTION trial (NCT03795298) will compare LAAC and OAC following AF ablation in patients suitable for OAC therapy while CLEARANCE (NCT04298723) and STROKECLOSE (NCT02830152) will evaluate the LAAC in patients with previous intracranial hemorrhage.

The OCCLUSION-AF (NCT03642509) trial seeks to assess the role of LAAC using an Amulet or Watchman device in patients at high risk of recurrent thromboembolic events and with a history of documented prior stroke or TIA, and who are eligible for OAC; this follows the results of the LAAOS III trial, which demonstrated the superiority of the surgical closure of the LAA when associated with OAC compared to OAC alone [52].

## 8. Conclusions

Watchman and Amulet devices offer excellent efficacy and safety in terms of LAAC for stroke prevention. No guidelines are currently available to choose one device over the other, and the device selection is based on center or physician preference and the level of comfort with each device system. Nevertheless, the patient’s LAA anatomy should be considered while selecting the Amulet or Watchman device for LAAC in consideration of the different designs and sealing mechanisms of the two devices.

## Figures and Tables

**Table 1:** Key findings of the LAAC randomized clinical trials. CI: confidence interval; CV: cardiovascular; DOAC: direct oral anticoagulant; HR: hazard ratio; HR: hazard ratio; LAAC: left atrial appendage closure; RR: risk ratio; SE: systemic embolism; sHR: subdistributional hazard ratio. TIA: transient ischemic attack; * Difference in event rates (Kaplan–Meier estimate or proportions) between groups. † These inclusion criteria were meant to include a higher risk group compared to PROTECT AF.

Trial	Design and Patient Selection	Patients	Key Findings
PROTECT AF	WATCHMAN vs. warfarinNon-inferiority Key Inclusion Criteria: Paroxysmal, persistent, or permanent nonvalvular AF; One or more CHADS_2_ risk factors; Eligibility for long-term anticoagulation with warfarin.	Control (*n* = 244) Device (*n* = 463)	Primary efficacy endpoint (stroke, SE, and CV/unexplained death) event rate: 3.0 per 100 patient-years (95% CI: 1.9–4.5) in the Watchman group and 4.9 per 100 patient-years (2.8–7.1) in the warfarin group (RR: 0.62, 95% CI: 0.35–1.25) Primary safety events: more frequent in the Watchman group (7.4 per 100 patient-years, 95% CI: 5.5–9.7 vs. 4.4 per 100 patient-years, 95% CI: 2.5–6.7; RR: 1.69, 1.01–3.19)
PREVAIL	WATCHMAN vs. warfarin Non-inferiority Key Inclusion Criteria: Paroxysmal, persistent, or permanent non-valvular AF; Eligible for long-term warfarin therapy; Calculated CHADS2 score of 2 or greater †.	Control (*n* = 138) Device (*n* = 269)	First coprimary endpoint of efficacy (stroke, SE, and CV/unexplained death) event rate: at 18 months was 0.064 with Watchman group vs. 0.063 in the warfarin group (RR 1.07, 95% CI: 0.57–1.89) Second coprimary endpoint of efficacy (stroke or SE after 7 days post-randomization) was 0.025 vs. 0.020 (risk difference 0.005, 95% CI: −0.019 to 0.027) Lower adverse events rate than PROTECT AF (4.2% vs. 8.7%)
PRAGUE-17	LAAC device vs. DOAC Non-inferiority Key Inclusion Criteria: Nonvalvular AF and one of the following: Bleeding requiring hospitalization or intervention; History of cardioembolic event while on anticoagulation; CHA_2_DS_2_-VASc score ≥ 3; HAS-BLED score ≥ 2.	Control (*n* = 201) Device (*n* = 201)	Annualized rate of the primary composite outcome (stroke, TIA, SE, CV death, major or non-major clinically relevant bleeding, or procedure-/device-related complications) was 10.99% with LAAC and 13.42% with DOAC (sHR 0.84; 95% CI: 0.53–1.31; *p* = 0.44; *p* = 0.004 for non-inferiority) Major LAAC-related complications occurred in 4.5% of patients
AMULET IDE	AMULET vs. WATCHMAN Non-inferiority Key Inclusion Criteria: Nonvalvular AF (paroxysmal, persistent, or permanent); CHA_2_DS_2_-VASc score of ≥3; Suitable for short-term OAC, but not suitable for long-term OAC.	Amulet (*n* = 934) Watchman (*n* = 944)	Primary safety endpoint at 12 months (procedure-related complications, major bleeding, non-procedure-related major bleeding, all-cause death) was 14.5% with Amulet and 14.7% with Watchman (HR = −0.14 *; 95% CI, −0.42 to 3.13) Primary effectiveness endpoint at 18 months (ischemic stroke, SE, major bleeding, primary mechanism of action endpoint) was 2.8% with Amulet vs. 2.8% with Watchman (HR = 0.00 *; 95% CI, −1.55 to 1.55) Composite outcome at 18 months (stroke, SE, CV/unexplained death) was 5.6% with Amulet vs. 7.7% with Watchman (HR = −2.12 *; 95% CI, −4.45 to 0.21)

**Table 2:** Device construction. LAA: left atrial appendage; PET: polyethylene terephthalate.

Parameter	Amulet	Watchman FLX	Watchman FLX Pro
Construct of the device	Self-expanding nitinol device with a distal lobe and a proximal disc	Self-expanding nitinol frame with PET with distal fluoroscopic marker	Self-expanding nitinol frame with HEMOCOAT technology covering
Anchor mechanism	Stabilizing wires	Dual-row anchors	Dual-row anchors with three radiopaque markers
Available sizes	8	5	6
Size range	16 mm, 18 mm, 20 mm, 22 mm, 25 mm, 28 mm, 31 mm, 34 mm	20 mm, 24 mm, 27 mm, 31 mm, 35 mm	20 mm, 24 mm, 27 mm, 31 mm, 35 mm, 40 mm
Ostium coverage	11–31 mm	14–31.5 mm	14–36 mm
Sealing mechanism	Dual seal mechanism (disc and lobe)	Single seal mechanism (single lobe)	Single seal mechanism (single lobe)
Access system	Amplatzer steerable sheath with the ability of bidirectional co-axial alignment allowing for 0–120° deflection.	Watchman TrueSeal (14 Fr) access system available in a single, anterior, and double curve; Watchman FXD (15 Fr) access system available in single and double curve; TrueSteer (17 Fr).	Watchman FXD (15 Fr) access system available in single and double curve; TrueSteer (17 Fr).

**Table 3:** Amulet and Watchman FLX release criteria.

Amulet	Watchman
Confirm proper device placement before release using echocardiography and fluoroscopy following CLOSE criteria C—At least 2/3 of the device lobe should be distal to the left Circumflex artery on echocardiography; L—The device Lobe should be slightly compressed and have good apposition to the left atrial appendage wall; O—The Orientation of the device lobe must be in line with the axis of the intended landing zone in the left atrial appendage; S—The disc must be Separated from the lobe; E—The disc will have a concave elliptical shape.	All criteria must be met prior to device release PASS criteria Position—Device is at the ostium of the LAA with the exclusion of all pectinate muscle; Anchor—Secure fixation anchors confirming device stability; Size—Device is compressed 10–30% of the original size; Seal—Effective sealing without peri-device leaks ledetected by color Doppler and/or angiographic assessment.

**Table 4:** Overview of current randomized trials on percutaneous LAAC with Amulet and Watchman devices. BARC: Bleeding Academic Research Consortium; CE: Conformite Europeenne; CV: cardiovascular; DOAC: direct oral anticoagulant; ICH: intracranial hemorrhage; LAAC: left atrial appendage closure; NI: non-inferiority; NVAF: nonvalvular atrial fibrillation; OAC: oral anticoagulation; S: superiority; SE: systemic embolization; TAVR: transcatheter aortic valve replacement; TIA: transient ischemic attack; VKA: vitamin K antagonist.

Trial	Sample	Objective	Intervention	Control	Primary Outcomes	Follow-Up
CHAMPION-AF (NCT04394546)	3000	Evaluate LAAC with Watchman/FLX in NVAF patients eligible for long-term DOAC	Watchman/FLX	Long-term DOAC	Composite endpoint of ischemic stroke or SE; composite endpoint of ischemic stroke, SE, or CV death (tested for NI); nonprocedural major bleeding (tested for S)	3 y
CATALYST (NCT04226547)	2650	Evaluate LAAC with Amulet in patients with NVAF eligible for long-term DOAC	Amulet	Long-term DOAC	Composite endpoint of ischemic stroke or SE; composite endpoint of ischemic stroke, SE, or CV death (tested for NI); nonprocedural major bleeding (tested for S)	3 y
OCCLUSION-AF (NCT03642509)	750	Evaluate Amulet or Watchman in patients with NVAF and prior ischemic stroke or TIA eligible for long-term DOAC	Amulet or Watchman	Long-term DOAC	Composite endpoint of stroke, SE, all-cause mortality, and major bleeding	5 y
CLOSURE-AF (NCT03463317)	1512	Evaluate LAAC in patients with NVAF at high bleeding risk or contraindication to OAC	LAAC devices with CE approval	DOAC or VKA	Composite endpoint of stroke, SE, CV, or unexplained death and major bleeding	2 y
STROKECLOSE (NCT02830152)	750	Evaluate LAAC in patients with NVAF and ICH within 12 months	Amulet	Medical therapy	Composite endpoint of stroke, SE, all-cause mortality, and major bleeding	5 y
CLEARANCE (NCT04298723)	550	Evaluate LAAC in NVAF patients with a previous ICH	Watchman FLX	Medical therapy	Composite endpoint of stroke, SE, CV, or unexplained death and major bleeding	2 y
COMPARE-LAAO (NCT04676880)	609	Evaluate LAAC in patients with NVAF and contraindications for OAC	Watchman FLX or Amulet	Antiplatelets or no therapy	Time to first stroke event; time to first stroke, TIA or SE event; procedural complications	5 y
OPTION (NCT03795298)	1600	Evaluate LAAC with Watchman FLX vs. OAC in patients with NVAF undergoing catheter ablation for NVAF	Watchman FLX	DOAC or VKA	Composite endpoint of stroke, SE, or death; no procedure-related major bleeding	3 y
ASAP-TOO (NCT02928497)	481	Evaluate LAAC in patients with NVAF and contraindications for OAC	Watchman	Single antiplatelet or no therapy	Time to the first occurrence of ischemic stroke and SE; 7-day combined rate of death, ischemic stroke, SE, and complications requiring major CV or endovascular intervention	5 y
LAAOS 4 (NCT05963698)	4000	Assess whether LAAC prevents ischemic stroke or systemic embolism in patients with NVAF, at high risk of stroke, despite receiving ongoing treatment with oral anticoagulation	Watchman plus OAC	OAC	Time to the first occurrence of ischemic stroke and SE	4 y
